# Targeted Methylation of the LncRNA NEAT1 Suppresses Malignancy of Renal Cell Carcinoma

**DOI:** 10.3389/fcell.2021.777349

**Published:** 2021-12-09

**Authors:** Jieqing Chen, Xinhui Liao, Jianli Cheng, Ganglin Su, Fen Yuan, Zhongfu Zhang, Jianting Wu, Hongbing Mei, Wanlong Tan

**Affiliations:** ^1^ Department of Urology, Nanfang Hospital, Southern Medical University, Guangzhou, China; ^2^ Department of Urology, Shenzhen Second People’s Hospital, The First Affiliated Hospital of Shenzhen University, Shenzhen, China; ^3^ Guangdong Key Laboratory of Systems Biology and Synthetic Biology for Urogenital Tumors, Shenzhen Second People’s Hospital, The First Affiliated Hospital of Shenzhen University, Shenzhen, China; ^4^ Shenzhen Key Laboratory of Genitourinary Tumor, Shenzhen Second People’s Hospital, The First Affiliated Hospital of Shenzhen University, Shenzhen, China

**Keywords:** lncRNA, NEAT1, m6A (N6-methyladenosine), RCC = renal cell cancer, dCas13b

## Abstract

Long-chain non-coding RNA (LncRNA) has been found to play an important role in the regulation of the occurrence and progression of renal cell carcinoma (RCC). In this study, we demonstrated that LncRNA NEAT1 expression and m6A methylation level was decreased in RCC tissues. Further, the downregulated expression level of LncRNA NEAT1 was associated with poor prognosis for RCC patients. Then we used CRIPSR/dCas13b-METTL3 to methylate LncRNA NEAT1 in RCC cells. The results showed that the expression level of LncRNA NEAT1 was upregulated after methylated by dCas13b-METTL3 in RCC cells. And the proliferation and migration ability of RCC cells was decreased after methylated LncRNA NEAT1. Finally, we examined the effect of LncRNA NEAT1 hypermethylation on the transcriptome. We found differentially expressed genes in RCC cells were associated with “cGMP-PKG signaling pathway”, “Cell adhesion molecules” and “Pathways in cancer”. In conclusion, CRISPR/Cas13b-METTL3 targeting LncRNA NEAT1 m6A methylation activates LncRNA NEAT1 expression and provides a new target for treatment of RCC.

## Introduction

Renal cell carcinoma (RCC) is a common malignant tumor of the urinary system and is the second leading cause of death among urinary system tumors. The occurrence of RCC is relatively hidden and difficult to detect, there are no typical clinical symptoms in the early stage, and there is no effective marker for early diagnosis of RCC (Cohen and McGovern, 2005). In addition, about 30% of patients have already developed metastasis when renal cancer is diagnosed. The rise of targeted drugs in the past decade has offered promise and improved survival benefits for patients with advanced kidney cancer, yet most of these patients develop resistance between 6 and 15 months (Schmitt and Chang, 2016). Therefore, it is important to further explore the underlying mechanisms of RCC pathogenesis and metastasis, and to find effective and reliable biomarkers for renal cancer progression and therapeutic targets.

Human genome sequencing found that more than 80% of the genes coding no proteins, these non-coding gene transcription of RNAs contain a kind of important long chain noncoding RNA (LncRNA), the non-coding RNA length of more than 200 nucleotides (Lin and Yang, 2018). Previous researches have observed that this kind of LncRNA in cells play an essential role in the process of various biology (Martens-Uzunova et al., 2014; Sun and Kraus, 2015). In addition, studies have shown that lncRNA plays an important role in the process of tumor genesis and progression, and has become the focus of attention of new tumor markers. (Sun and Kraus, 2015).

In recent studies, with the use of RNA-seq, many LncRNAs related to RCC have been discovered. For example, miRNA-141 reduces the proliferation and invasion ability of RCC cells by regulating lncRNA HOTAIR, thus hindering the progression and metastasis of renal cancer (Chiyomaru et al., 2014). Hirata et al. reported that MALATL could promote the invasion of RCC by binding EZH2, and this binding was regulated by miR-205 (Hirata et al., 2015). LncRNA NEAT1 is transcribed from a gene locus on chromosome 11 known as multiple endocrine tumor type 1 of familial tumor syndrome, and is a long-chain non coding RNA encoding two variants of NEAT1_v1 (3.7 kb) and NEAT1_v2 (23 kb) (Naganuma and Hirose, 2013). The expression of LncRNA NEAT1 has been found abnormal in many malignant tumors, including glioma (He et al., 2016), human laryngeal squamous cell carcinoma (Wang et al., 2016), gastric cancer (Choudhry et al., 2015), breast cancer (Fu et al., 2016), esophageal cancer (Chen et al., 2015), etc. These studies suggest that the abnormal expression of lncRNA NEAT1 is a key regulator of tumor genesis and progression, and can be used as a potential target for therapeutic intervention.

In the study of this subject, we screened and analyzed multiple databases and found the LncRNA NEAT1 with obvious differential expression in RCC tumor tissues compared to normal tissues. And, the low m6A modification of LncRNA NEAT1 in RCC was also verified. Subsequent mechanism studies have shown that CRISPR/dCas13b-METTL3 methylates LncRNA NEAT1 through m6A modification, thereby promoting the cells proliferation and metastasis. This study confirmed the effect of LncRNA NEAT1 for inhibiting the progression and migration of RCC, and might provide a new molecular marker for the diagnosis and treatment of RCC.

## Results

### The mRNA and m6A Methylation Level of LncRNA NEAT1 Was Significantly Decreased in RCC Tumor Tissues

**FIGURE 1:**
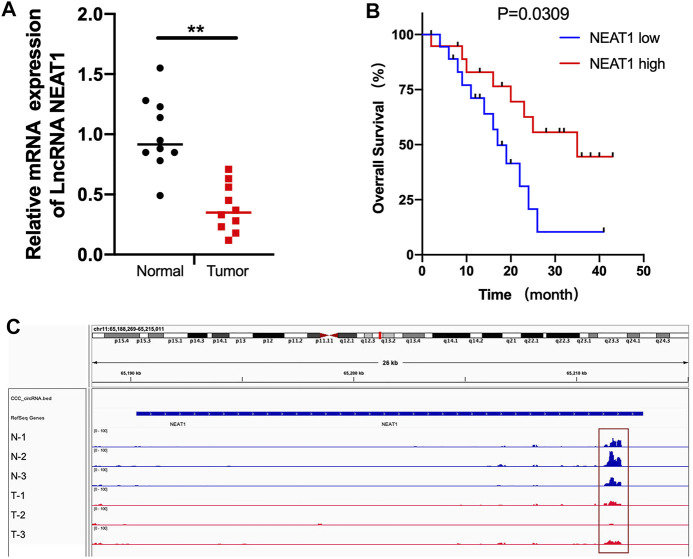
The mRNA and m6A methylation level of LncRNA NEAT1 was obviously decreased in RCC tissues. **(A)** LncRNA NEAT1 expression levels in RCC tumors and normal tissues. **(B)** The Kaplan–Meier survival curves of LncRNA NEAT1 in RCC tissues. **(C)** The m6A abundances on LncRNA NEAT1 exon in 3 pairs of RCC tumor and adjacent tissues by MeRIP-seq.

First, we detected the mRNA level of LncRNA NEAT1 in 10 pairs of RCC tumors and normal tissues. As shown in Figure 1A, LncRNA NEAT1 was obviously downregulated in tumor tissues. In addition, we observed that RCC patients with low expression of LncRNA NEAT1 had a poor prognosis compared with patients with high expression of LncRNA NEAT1 (Figure 1B). From our previous MeRIP-seq data, we found LncRNA NEAT1 had a higher m6A modification in adjacent normal tissues than that in RCC tissues (Figure 1C). In conclusion, our data exhibited that LncRNA NEAT1 might played an important function for the tumorigenesis of RCC.

### CRIPSR/dCas13b-METTL3 Increased the Expression of LncRNA NEAT1 in RCC Cell Lines

**FIGURE 2:**
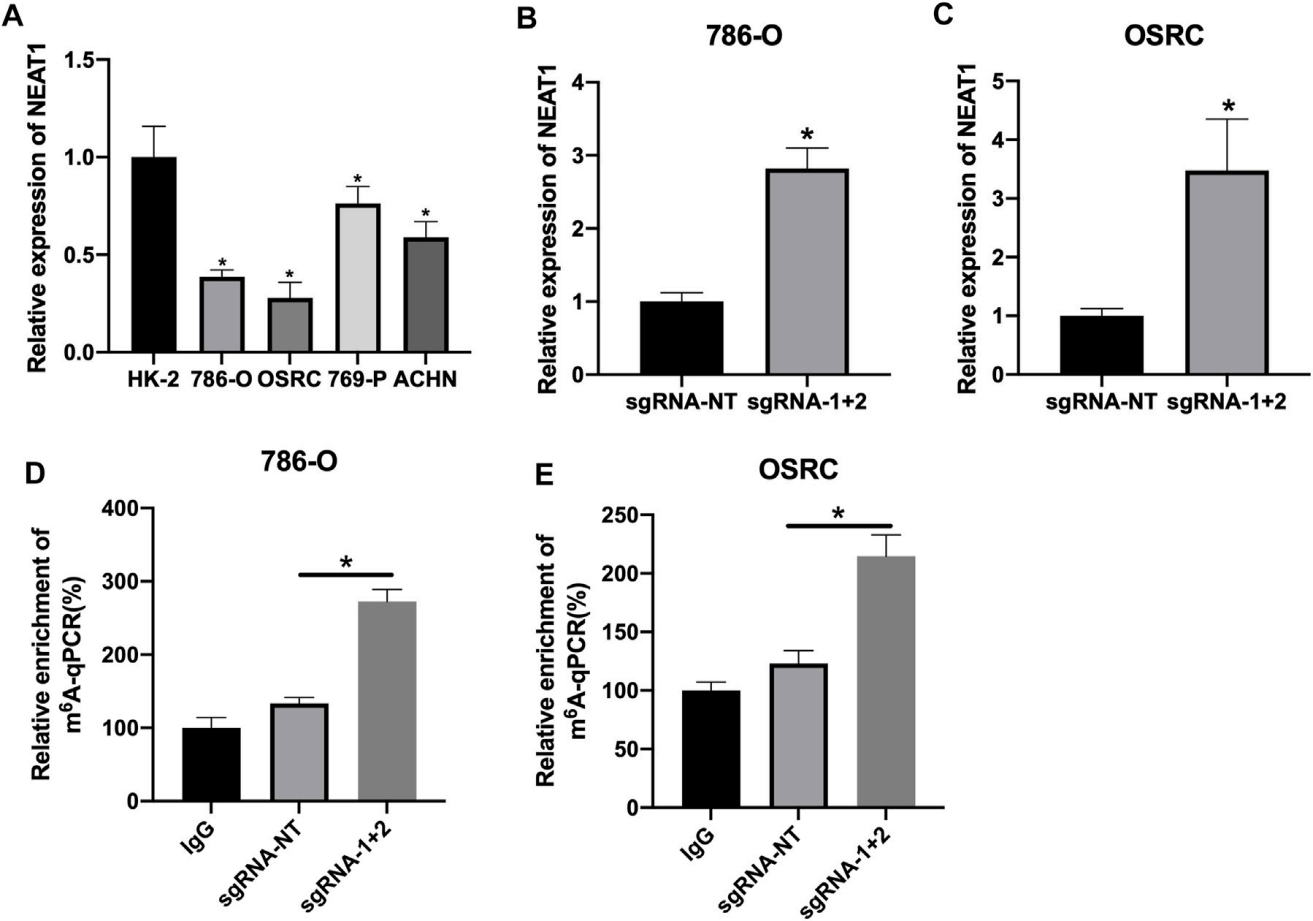
CRIPSR/dCas13b-METTL3 increased the expression level of LncRNA NEAT1 in RCC cells**. (A)** The mRNA levels of LncRNA NEAT1 in RCC cell lines (786-O, 769-P, ACHN and OSRC) and normal renal cell (HK2) detected by RT-qPCR. **(B,C)** The mRNA levels of LncRNA NEAT1 in 786-O cells **(B)** and OSRC cells **(C)** after transfected with dCas13b-METTL3 and sgRNAs for 24 h detected by RT-qPCR. **(D,E)** The m6A levels of LncRNA NEAT1 in 786-O cells **(D)** and OSRC cells **(E)** after transfected with dCas13b-METTL3 and sgRNAs for 24 h detected by MeRIP-qPCR. * *p* < 0.05.

The mRNA levels of LncRNA NEAT1 in various RCC cells was detected. We observed that LncRNA NEAT1 was downregulated in RCC cells (786-O, 769-P, OSRC and ACHN) compared to normal renal cell line (HK2) (Figure 2A). Another research had confirmed CRIPSR/dCas13b-METTL3 could increase the m6A methylation level by m6A RNA modification (Wilson et al., 2020). Then we transfected with dCas13b-METTL3 fusion protein in 786-O and OSRC cells, and found LncRNA NEAT1 was increased when transfected with dCas13b-METTL3 and sgRNAs for LncRNA NEAT1 compared to control (Figures 2B,C).

### Hypermethylation of LncRNA NEAT1 Represses the RCC Cells Proliferation

**FIGURE 3:**
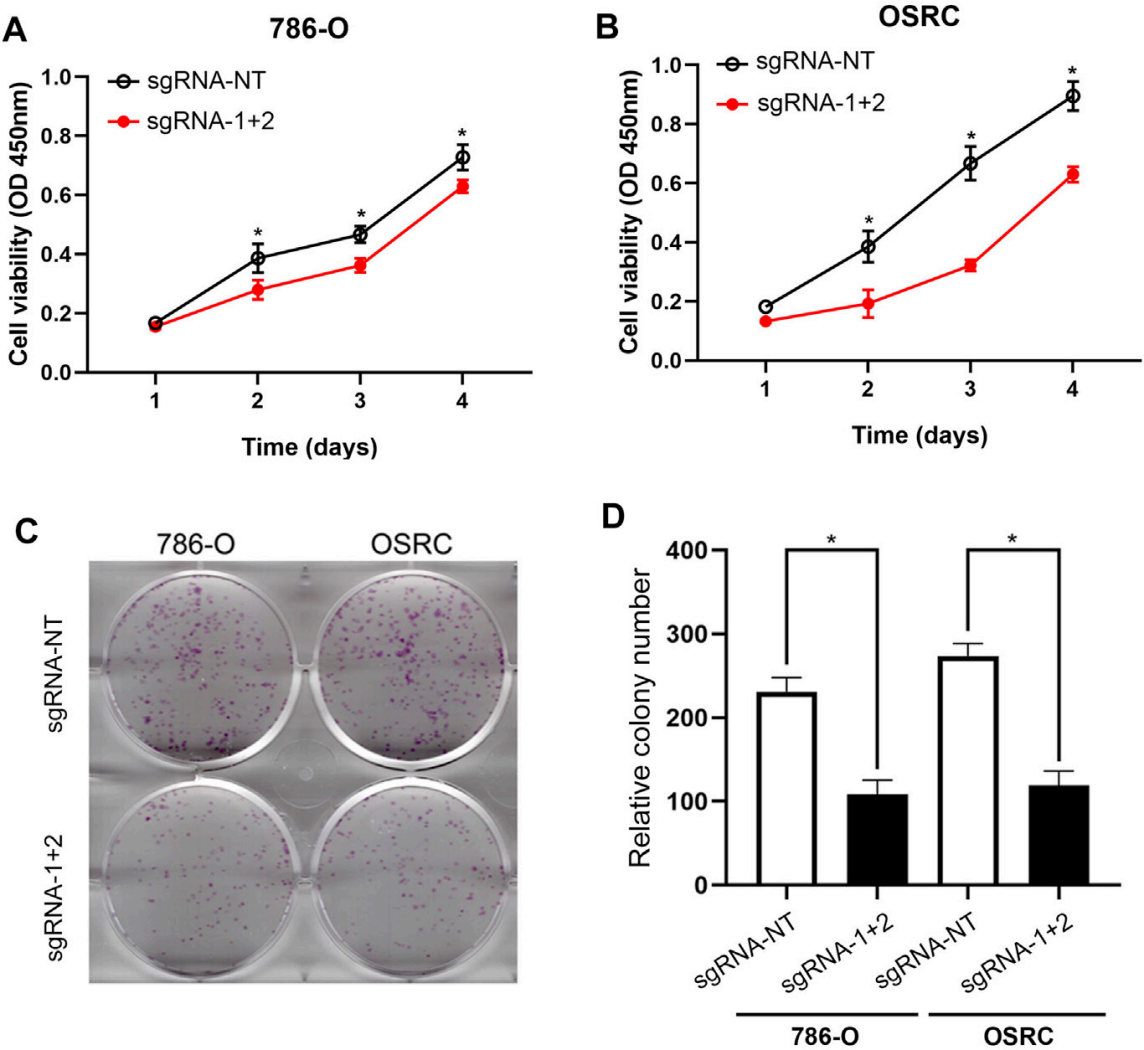
Hypermethylation of LncRNA NEAT1 repressed the RCC cells proliferation. **(A,B)** CCK-8 assays of 786-O and OSRC cells after transfected with dCas13b-METTL3 and sgRNAs. **(C,D)** Colony formation assay for 786-O and OSRC cells after transfected with dCas13b-METTL3 and sgRNAs. *p < 0.05.

Further, we detected if the hypermethylation of LncRNA NEAT1 repressed RCC cells proliferation. The results of CCK-8 assay showed that the hypermethylation of LncRNA NEAT1 in RCC cells obviously repressed cells proliferation (Figures 3A,B). In addition, we performed the colony formation assay and found the LncRNA NEAT1 hypermethylated 786-O and OSRC cells significantly inhibited the cell growth compared to the controls (Figures 3C,D). These results showed that hypermethylated LncRNA NEAT1 by dCas13b-METTL3 inhibited the proliferation of RCC cells.

### Hypermethylation of LncRNA NEAT1repressed the RCC Cells Migration

**FIGURE 4:**
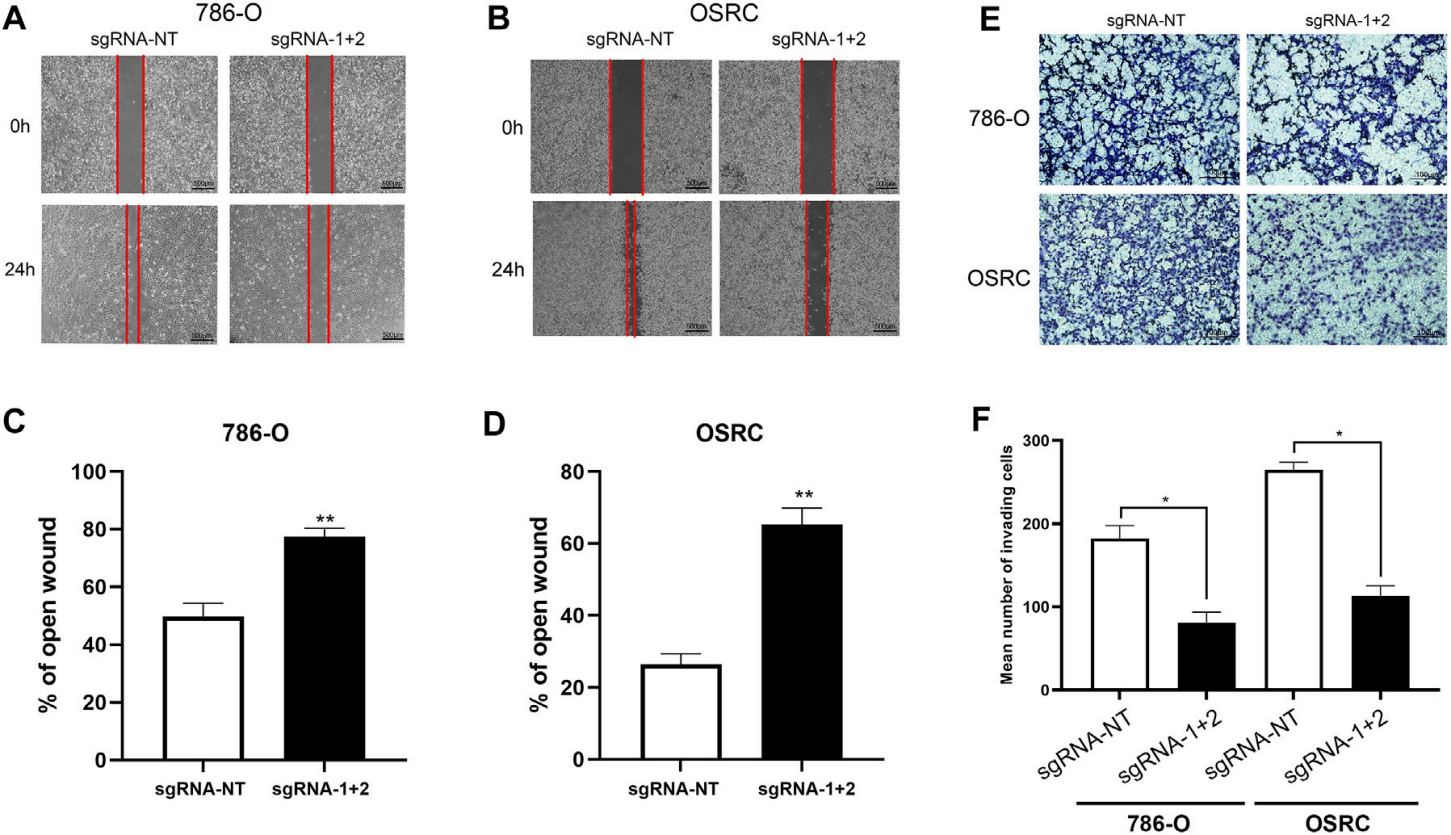
Hypermethylation of LncRNA NEAT1 repressed the RCC cells migration. **(A-D)** Wound-healing assays for migration ability of 786-O and OSRC cells after transfected with dCas13b-METTL3 and sgRNAs. **(E,F)** Transwell assays for migration ability of 786-O and OSRC cells after transfected with dCas13b-METTL3 and sgRNAs. *p < 0.05.

Next, we determined the RCC cells migration after hypermethylated LncRNA NEAT1. The results of wound-healing assays and transwell assays exhibited that LncRNA NEAT1 hypermethylated 786-O and OSRC cells had a decreased migration ability compared to that in the control cells (Figures 4A–F). These data showed that hypermethylated LncRNA NEAT1 by dCas13b-METTL3 repressed the migration of RCC cells.

### Transcriptional Regulation by Hypermethylation of LncRNA NEAT1

**FIGURE 5:**
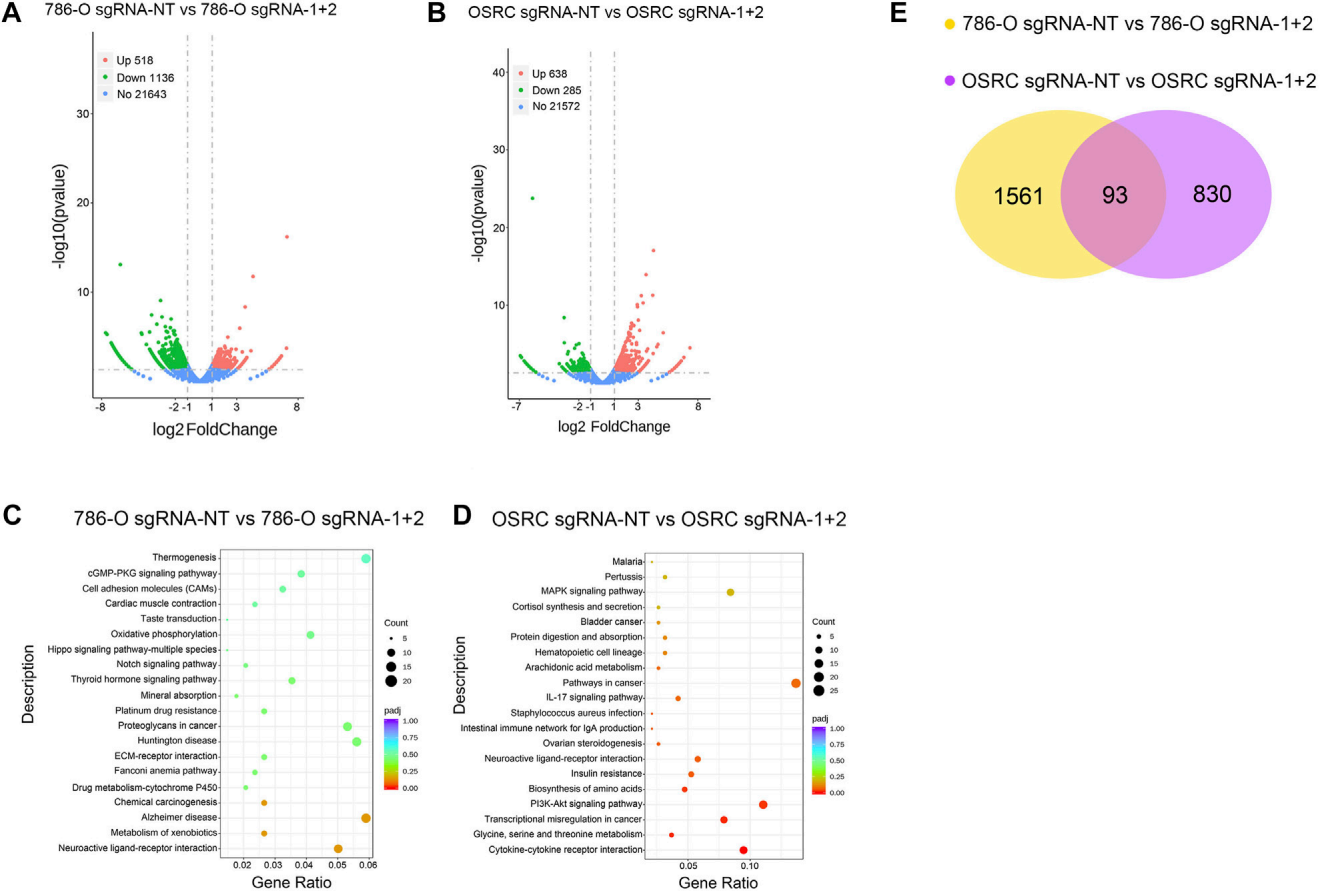
The effect of LncRNA NEAT1 Hypermethylation on the transcriptome **(A,B)** Differentially expressed genes in 786-O and OSRC cells after transfected with dCas13 b-METTL3 and sgRNAs **(C,D)** KEGG pathway analysis of differentially expressed genes in 786-O and OSRC cells **(E)** The Venn diagram summarizes the overlap of differentially expressed genes in both cells.

Finally, we determined the effect of LncRNA NEAT1 hypermethylation on the transcriptome of RCC cells by high throughput RNA sequencing. The results showed that there are 1,654 and 923 genes exhibited differentially expressed levels in LncRNA NEAT1 hypermethylated 786-O and OSRC cells compared to control cells (Figures 5A,B). Then we analyzed the changes of the KEGG pathway and found differential expressed genes were mostly related to “cGMP-PKG signaling pathway”, “Cell adhesion molecules”, “Hippo signaling pathway-multiple species”, “Notch signaling pathway”, “MAPK signaling pathway”, “Pathways in cancer”, “PI3K-Akt signaling pathway” and “Transcriptional misregulation in cancer” in 2 cell lines (Figures 5C,D). By comparing differentially expressed genes, we identified 93 genes that are regulated by LncRNA NEAT1 in both cells (Figure 5E). These results demonstrated that LncRNA NEAT1 might be a transcriptional regulator which could regulate the occurrence and development of RCC.

## Discussion

Renal cell carcinoma (RCC) is a common malignancy of the genitourinary system, accounting for a high proportion of adult malignancies. In addition, the incidence of RCC is increasing year by year globally, and there have been reports of a significant increase in the death rate of RCC, especially among people aged 60 and older. The specific etiology of RCC is not clear, but the incidence of RCC has been found to be related to smoking, genetics, obesity, hypertension and other factors (Cohen and McGovern, 2005). In clinical practice, the onset of RCC is often insidious and the early clinical manifestations are often absent. Most RCC patients are found by physical examination without conscious symptoms. These patients account for more than 50–60% of RCC patients, and nearly 30% of kidney cancer patients have metastases at the time of diagnosis. Therefore, it is urgent to find out the molecular mechanism of RCC pathogenesis and explore molecular targets for early diagnosis, treatment and prognosis evaluation of RCC.

Non-coding RNA is a kind of non-protein-coding RNA, which can be divided into miRNA and lncRNA according to the number of its nucleotides. LncRNA is a new type of non-coding RNA that has attracted much attention in recent years (Bao et al., 2017). Previous studies have shown that the abnormal expression of many LncRNAs, such as LOC653786, ROR, GIHCG and PVT1 are correlated with the occurrence and development of RCC (Bao et al., 2017; Yang et al., 2018; D'Aniello et al., 2018; Ren et al., 2019; Shi et al., 2019). NEAT1 plays a regulatory role in many tumors, but there are few reports on the role of NEAT1 in RCC.

In our study, we confirmed that the expression of LncRNA NEAT1 was decreased in RCC tumors and RCC cells, which indicated LncRNA NEAT1 might be related to the occurrence and progression of RCC. The MeRIP-seq data of five pairs of RCC tissues showed that the m6A modification of LncRNA NEAT1 was hypomethylated in RCC tumors. Then we performed CRIPSR/dCas13b-METTL3 to methylate LncRNA NEAT1 in two kinds of RCC cells. The results showed that the expression of LncRNA NEAT1 were upregulated after hypermethylated by dCas13b-METTL3 in 786-O and OSRC RCC cells. In addition, the RCC cells proliferation and migration ability was detected by CCK8 and scarification assays. The results showed that the RCC cells transfected with dCas13b-METTL3 and sgRNA for LncRNA NEAT1 exhibited the decreased proliferation and migration ability compared to control cells. Finally, we examined the change of transcription level in LncRNA NEAT1 hypermethylated RCC cells. The results showed that there were 1,654 and 923 differentially expressed genes in LncRNA NEAT1 hypermethylated 786-O and OSRC cells compared to control cells, and found these differential expressed genes were associated with “cGMP-PKG signaling pathway”, “Cell adhesion molecules” and “Pathways in cancer”.

Collectively, the expression and m6A methylation level of LncRNA NEAT1 is frequently decreased in RCC. CRISPR/Cas13b-METTL3 targeting LncRNA NEAT1 m6A methylation activates LncRNA NEAT1 expression and suggested its potent tumor-suppressive function. We believe that our findings can provide a new target for the treatment of RCC.

## Materials and Methods

### Cell Lines Culture and Plasmid Transfection

Both renal cancer cell lines (786-O, 769-P, OSRC and ACHN) and normal renal cell line (HK2) were purchased from Shanghai Cell Bank, Chinese Academy of Sciences. Renal tubule epithelial HK-2 cells were cultured with high glucose medium containing 10% FBS DMEM, and renal carcinoma cells ACHN, 786-O, 769-P and OSRC were cultured with RPMI 1640 medium containing 10% FBS. Culture and passage in 37°C, 5% CO2 incubator. Cells at logarithmic growth stage were inoculated in 6-well plates, and transfected according to the Lipofectamine 3,000 transfection reagent instructions after the degree of cell fusion reached 50%. The plasmid was replaced with fresh medium.

### RNA Isolation

Trizol (Invitrogen, USA) was added to cells and placed in a mortar. The grinding process is carried out on ice until the specimen is a thick liquid. After 10 min at room temperature, the specimens were moved into EP tubes respectively, and chloroform was added and violently shaken for mixing. After 10 min at room temperature, the specimens were centrifuged. Finally, the precipitation was washed with 75% ethanol. Add DEPC water to dissolve and precipitate and determine the concentration.

### Reverse Transcription and RT-qPCR

RNA was reverse transcribed into cDNA using the PrimeScript RT Master kit (Takara, RR037A). RT-qPCR was performed with the SYBR® Premix EX TaqTM II PCR Kit (Takara, DRR041A) following the manufacturer’s instructions on the Roche Lightcycler 480 Real-Time PCR System. Data were calculated according to the Applied Biosystems comparative Ct method. Primers used for LncRNA NEAT1 and GAPDH qRT-PCR were as follows:

LncRNA NEAT1-Forward: 5′-GGA​TGA​GGC​CTG​GTC​TTG​T-3’.

LncRNA NEAT1-Reverse: 5′-GAG​AAA​AGT​CCA​AAA​GGA​GCA​C-3’.

GAPDH-Forward: 5′-ACA​ACT​TTG​GTA​TCG​TGG​AAG​G-3’.

GAPDH-Reverse:5′-GCCATCACGCCACAGTTTC-3’.

### CCK-8 Assay

RCC cells in each group were inoculated in 96-well plates at a density of 3×10^3^/well, and 150 μl of medium was added to each well. On day 1, 2, 3, 4 and 5 after inoculation, 4 wells were taken from each group, and 15 μl of (5 mg/L) A solution (BD Biosciences, Bedford, MA) was added to each well, and the cells were cultured in an incubator for 3 h. Add B solution 150 μl to each well, and vibrate at low speed for 10 min. The absorbance (A) value of each well at the wavelength of 490 nm was detected by the enzyme plate analyzer, and the average value of the 4 wells was taken. The cell growth curve was drawn with time as the horizontal axis and absorbance A as the vertical axis.

### Transwell Assay

The Matrigel glue was diluted with serum-free medium, then spread 20 μl Matrigel glue in the upper chamber of Transwell, and placed in the incubator for polymerization for 2 h. Cells were collected from each group after infection, the cell concentration was adjusted to 1 × 10^5^ cells/mL, and 200 μl cell suspension was added into the upper compartment, and 600 μl medium containing 10% serum was added into the lower compartment. After cultured in the incubator for 24 h, the cells were taken out and stained with 0.1% crystal violet for 20 min. The dye was washed with phosphate buffer solution and the upper Matrigel gel and cells were wiped off with cotton swab. At high magnification, four fields were randomly selected for counting, and the number of transmembrane cells represented the invasion ability of RCC cells.

### Wound-Healing Assay

First, use marker to evenly draw a horizontal line behind the 6-hole plate. About 5 × 105 cells were added to the well. The next day, scratch the cell layer with the head of the gun, perpendicular to the cell plane, along the line drawn on the back of the plate the day before. After the scratches were completed, sterile PBS was used to wash the cells for 3 times, and the cells that did not adhere to the wall were washed away. Then replace the fresh serum-free medium. The cells were cultured in a 5% CO2 incubator at 37°C. The cells were then taken out at the appropriate point in time, and the width of the scratches was measured and photographed under a microscope line. After opening the Image with Image J software, randomly draw 6 to 8 horizontal lines to calculate the mean distance between cells.

### Colony Formation Assay

Trypsin digested the adherent cells, prepared cell suspension, counted cells, and adjusted the concentration to 1 × 103 cells/mL. Add 200 cells to each 6-well plate, add medium to 4 ml, shake thoroughly, place in the incubator for about 2 weeks, change solution every 2–3 days. Cells were observed regularly. When more than 100 cell clusters appeared, cell culture was stopped, the culture medium was sucked, washed with PBS for 3 times and then sucked away, and an appropriate amount of polymethanol was added for fixation for 20 min. The formaldehyde solution was discarded, and then an appropriate amount of crystal violet solution (0.1%) was added, and the stain was left standing for 30 min. The crystal violet solution was absorbed and discarded, and then the background color was fully washed with PBS, and the hole plate was fully dried and put on the white paper, and the photo was taken for counting.

### RNA-Seq & Data Analysis

Total RNA was extracted from biological samples using Trizol Regeant (Life Technologies) according to Manufacturer’s protocol. Denatured agarose gel electrophoresis was applied to evaluate integrity of total RNA. Seq-Star TM poly(A) mRNA Isolation Kit (Arraystar, MD, USA) was used to purify mRNA from total RNA after confirming its quantity and quality by NanoDrop ND-2000. Then fragmented mRNA was subjected to 50-bp single-end sequencing with a BGISEQ-500 platform. Adapter and low-quality reads were trimmed by SOAPnuke, and trimmed reads were aligned to reference genome by bowtie2. RSEM was used to calculate expression levels, and DEGs were identified by DEGseq.

### Enrichment Analysis of KEGG Pathway of Differentially Expressed Genes

Enrichment analysis of KEGG differentially expressed genes was performed using the cluster analysis Profiler R package, which corrected for gene length bias. KEGG Pathways with corrected *p* < 0.05 (FDR <0.05) were considered to be significantly enriched in differentially expressed genes. In addition, the author also used gene MANIA in Cytoscape3.6 to reveal the interaction network between KEGG pathway and genes in GO functional annotation.

### Statistical Analysis

All data were statistically analyzed by SPSS19.0 software. The measurement data were expressed as X ± S, and the comparison between the mean values of the two samples was conducted by two independent samples T test, and *p* < 0.05 was considered as statistically significant difference.

## Data Availability

The datasets presented in this study can be found in online repositories. The names of the repository/repositories and accession number(s) can be found below: https://www.ncbi.nlm.nih.gov/, PRJNA719079.
